# Development and Validation of a Mathematical Model to Predict the Complexity of *FMR1* Allele Combinations

**DOI:** 10.3389/fgene.2020.557147

**Published:** 2020-11-13

**Authors:** Bárbara Rodrigues, Emídio Vale-Fernandes, Nuno Maia, Flávia Santos, Isabel Marques, Rosário Santos, António J. A. Nogueira, Paula Jorge

**Affiliations:** ^1^Molecular Genetics Unit, Centro de Genética Médica Dr. Jacinto Magalhães (CGMJM), Centro Hospitalar Universitário do Porto (CHUP), Porto, Portugal; ^2^Unit for Multidisciplinary Research in Biomedicine (UMIB), Institute of Biomedical Sciences Abel Salazar (ICBAS), University of Porto, Porto, Portugal; ^3^Centre for Medically Assisted Procreation/Public Gamete Bank, Centro Materno-Infantil do Norte Dr. Albino Aroso (CMIN), Centro Hospitalar Universitário do Porto (CHUP), Porto, Portugal; ^4^Center for Environmental and Marine Studies (CESAM), Department of Biology, University of Aveiro, Aveiro, Portugal

**Keywords:** *FMR1* gene, CGG repeats, AGG interspersion pattern, modeling allelic complexity, *allelic score*

## Abstract

The polymorphic trinucleotide repetitive region in the *FMR1* gene 5′UTR contains AGG interspersions, particularly in normal-sized alleles (CGG < 45). In this range repetitive stretches are typically interrupted once or twice, although alleles without or with three or more AGG interspersions can also be observed. AGG interspersions together with the total length of the repetitive region confer stability and hinder expansion to pathogenic ranges: either premutation (55 < CGG < 200) or full mutation (CGG > 200). The AGG interspersions have long been identified as one of the most important features of *FMR1* repeat stability, being particularly important to determine expansion risk estimates in female premutation carriers. We sought to compute the combined AGG interspersion numbers and patterns, aiming to define *FMR1* repetitive tract complexity combinations. A mathematical model, the first to compute this cumulative effect, was developed and validated using data from 131 young and healthy females. Plotting of their allelic complexity enabled the identification of two statistically distinct groups – *equivalent* and *dissimilar* allelic combinations. The outcome, a numerical parameter designated *allelic score*, depicts the repeat substructure of each allele, measuring the allelic complexity of the *FMR1* gene including the AGGs burden, thus allowing new behavioral scrutiny of normal-sized alleles in females.

## Introduction

The fragile X-related disorders result from the expansion of a CGG-repeat tract in the 5′ untranslated region of the *FMR1* gene (Xq27.3), coding for the fragile X mental retardation protein (FMRP), an RNA-binding protein that regulates expression of several genes (Man et al., 2017). Depending on the number of CGG repeats, *FMR1* alleles can be categorized into four classes: normal (CGG < 45), intermediate or “gray zone” (45 < CGG < 54), premutation (55 < CGG < 200), and full mutation (CGG > 200) (Biancalana et al., 2015). Premutations causing *FMR1* mRNA overexpression and reduced FMRP synthesis, underly both fragile X-associated tremor/ataxia syndrome (FXTAS, OMIM #300623) and fragile X-associated primary ovarian insufficiency (FXPOI, OMIM #311360). The full mutation alleles undergo hypermethylation, leading to gene silencing and absence of FMRP, causing fragile X syndrome (FXS, OMIM #300624), the most common heritable cause of intellectual disability (Man et al., 2017). Due to the repeat tract instability, above a threshold expansions and contractions can be observed both in the germline and in the somatic cells. Some rare contraction events can originate mosaicism with mutated and normal alleles in clinically typical fragile-X phenotypes (Maia et al., 2016). In the normal population, the vast majority of the alleles contain one or more AGG interspersions within the repetitive tract, usually at every 9 or 10 CGG repeat intervals, being highly stable. In higher repeat ranges, the number of AGGs tends to be progressively smaller as the size of the repetitive tract increases (Yrigollen et al., 2012; Mila et al., 2018; Manor et al., 2019). The AGG interspersions together with the repetitive region’s total length confer stability and hinder expansion to pathogenic size-ranges (Latham et al., 2014; Domniz et al., 2018; McGinty and Mirkin, 2018). In premutation female carriers, the risk of having a child with FXS depends on both the repeat length and AGG interspersions (Ardui et al., 2018). The incidence of normal pure alleles (without interspersions) is low and their origin as well as the phenotypic impact in females, are still debatable. It has been proposed that “low zone” alleles, variably determined to be CGG ≤ 26 or CGG ≤ 23, are associated with different phenotypic outcomes (Mailick et al., 2014; Gleicher et al., 2015; Rehnitz et al., 2018). Some studies show that they are associated with decreased ovarian reserve and fertility issues, due to a mechanism not yet elucidated, possibly different from that involved in premutated alleles (Gleicher et al., 2015; Wang et al., 2017), although such negative effects were not corroborated by others (Spitzer et al., 2012; Ruth et al., 2016). These contradictory assumptions require further studies to elucidate the clinical impact of “low zone” alleles.

Few studies focus on AGG interspersion patterns to assess allele stability, within the normal range. Given the importance of understanding the cumulative effect of the CGG repeat tract length and its AGG interspersions, we developed a mathematical model that considers these patterns and produces a functional model predicting the complexity of allele combinations (*allelic score*).

## Materials and Methods

### Study Population

Young and potentially fertile females were recruited among candidates for oocyte donation at the Portuguese Public Gamete Bank, Centro Materno-Infantil do Norte Dr. Albino Aroso (CMIN), Centro Hospitalar Universitário do Porto (CHUP). The donor population, originating from the entire national territory, includes actively recruited students from major Portuguese universities, with a wide range of nationalities. Around 10% of the donor candidates were of foreign nationality, 95% were Caucasian and about 30% of those who donated at our center lived outside Porto (Galvão et al., 2017). Two independent cohorts were used for development (cohort 1) and for validation (cohort 2) studies. Cohort 1, *n* = 50, mean age 25.4 ± 3.93 years (range 18–33), recruited between 2016 and 2017. Cohort 2, *n* = 81, mean age 26.5 ± 3.86 years (range 19–33), collected between 2018 and 2019. All participants provided written informed consent, and this project was approved by the Hospital’s Ethics Committee (2018.231/201-DEFI/200-CES).

### *FMR1* Repeat Region Substructure Profile

Sizing of *FMR1* alleles had been previously obtained as part of the routine oocyte donor’s protocol, on blood samples. Categorizing the respective genotype followed the ACMG/EMQN guidelines: normal (CGG < 45), intermediate or “gray zone” (45 < CGG < 54), premutation (55 < CGG < 200), and full mutation (CGG > 200) (Monaghan et al., 2013; Biancalana et al., 2015). AGG interspersion pattern was determined by Triplet Repeat Primed-PCR using FRAXA PCR kit LabGscan^TM^ (Diagnostica Longwood, Zaragoza, Spain), according to the manufacturer’s instructions. This method allowed the confirmation of the total repeat length and the characterization of the CGG/AGG substructure. Thirteen samples with different patterns were additionally verified by Sanger sequencing to confirm the previously determined CGG/AGG pattern.

### Statistical Analysis

Hierarchical Cluster Analysis using euclidean distance as a metric to evaluate similarity was used in statistical software SPSS^®^ version 26 (IBM developer, 2019: SPSS Statistics version 26 – Armonk, New York, United States). Linear regression of the linearized form of an exponential model [i.e., regression of ln(score 2) against score 1] was used to obtain a functional model to relate the complexity of both alleles in each sample. The analysis of covariance (ANCOVA), as outlined by Zar (2010), was used to compare the regression models, and derive common regression lines, with *allelic scores* as variables [i.e., score 1 and ln(score 2)]. All statistical tests were carried out for a significance level of 0.05.

### Determination of X-Chromosome Inactivation Pattern and *FMR1* Methylation Status

X-chromosome inactivation (XCI) pattern was determined by the human androgen-receptor assay (HUMARA), resorting to the CAG trinucleotide repeat located in the first exon and two methylation-sensitive endonuclease sites located upstream of the *AR* gene (Allen et al., 1992). The percentage of allele activity was determined using the peak heights, and normalized to the corresponding undigested allele peak height. The *FMR1* methylation status was determined using AmplideX^®^ mPCR *FMR1* kit (Asuragen, Inc., Austin, TX, United States), according to the manufacturer’s instructions. The mPCR assay determines both the number of CGG repeats and the percentage promoter methylation of each *FMR1* allele.

## Results

A similar *FMR1* CGG size distribution was obtained in both cohorts with normal alleles, ranging from 15 to 40 CGG in cohort 1 and from 15 to 44 CGG in cohort 2 (*n* = 127, 97%) and intermediate genotypes, one allele with 48 CGG in cohort 1 and three alleles with 45 CGG in cohort 2 (*n* = 4, 3%) (Tables 1, 2). Homozygosity was observed in eleven samples (22%, cohort 1), of which nine shared the same CGG/AGG substructure, and in seventeen samples (21%, cohort 2), of which thirteen shared the same AGG pattern. In line with previous publications, the vast majority of the alleles (93%) showed one or two AGGs, 5% were pure (4, cohort 1 and 9, cohort 2) and the remaining 2% showed three AGG interspersions. The most common structure, (CGG)_10_AGG(CGG)_9_AGG(CGG)_9_, was identified in 29 (29%, cohort 1) and 40 alleles (25%, cohort 2). Similar to other worldwide populations, a highly polymorphic CGG/AGG substructure was observed: forty-one and fifty-five unique patterns were identified in cohorts 1 and 2, respectively (Tables 1, 2; Yrigollen et al., 2014).

**TABLE 1:** Cohort 1 data used to calculate the *allelic scores*, and identify the two groups, *equivalent* (white background) and *dissimilar* (gray background).

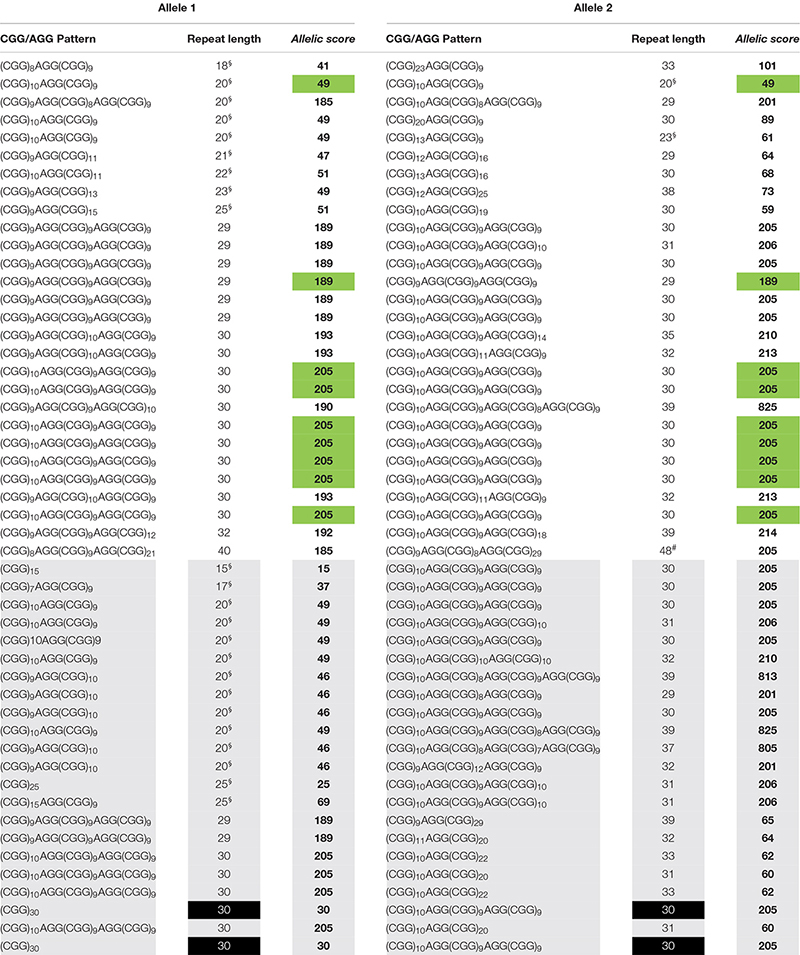

*Homoallelism for CGG-repeat length (black background) and homozygosity for both CGG-repeat length and AGG pattern (*allelic score* in green background).*

*^#^intermediate size.*

*^§^ normal “low zone” alleles (see section “Discussion”).*

**TABLE 2:** Cohort 2 data used to calculate the *allelic scores*, and identify the two groups, *equivalent* (white background) and *dissimilar* (gray background).

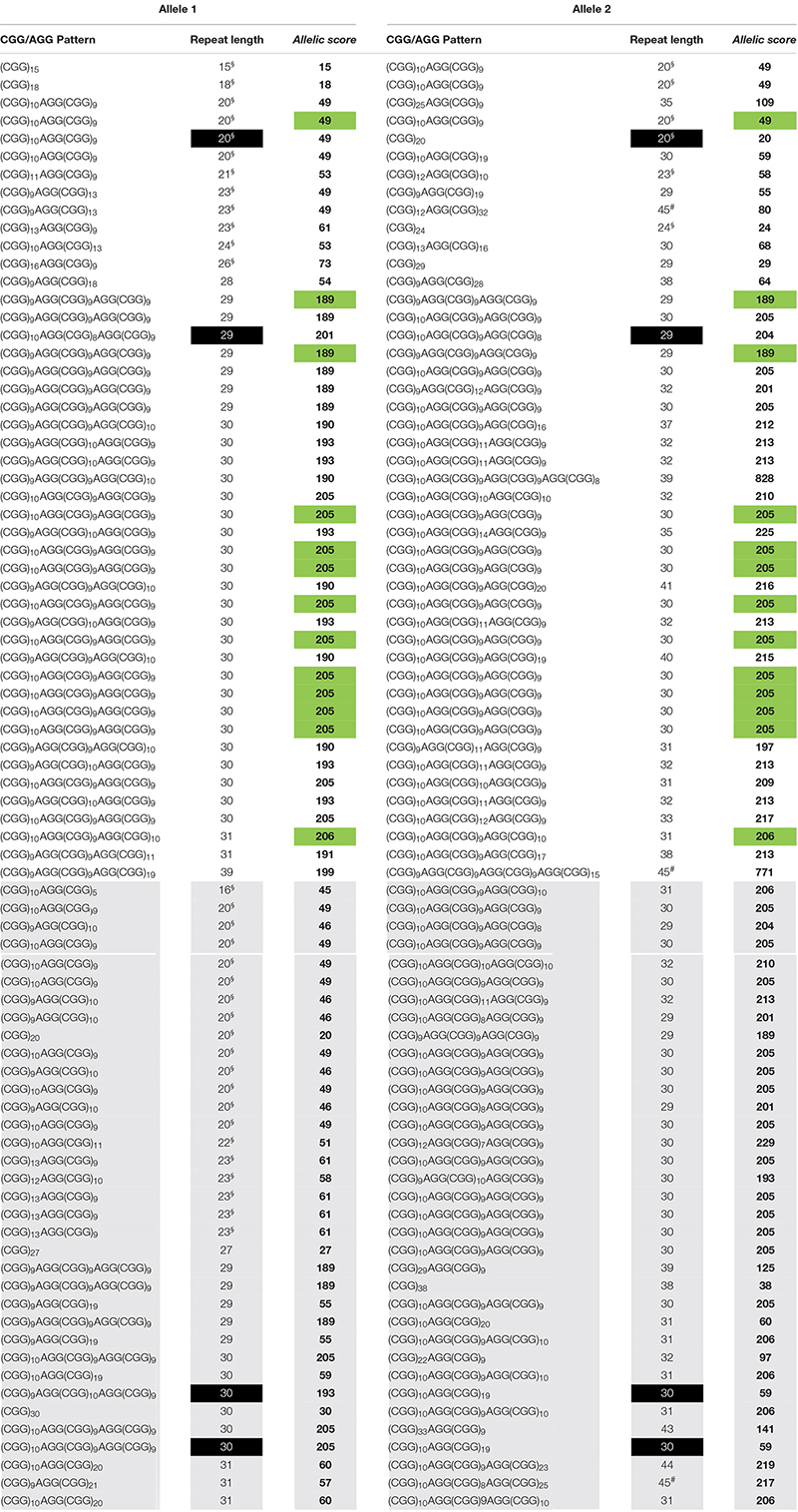

*Homoallelism for CGG-repeat length (black background) and homozygosity for both CGG-repeat length and AGG pattern (*allelic score* in green background).*

*^#^intermediate size.*

*^§^normal “low zone” alleles (see section “Discussion”).*

### Development of the Mathematical Model

A mathematical model was developed to integrate the AGG interspersion number and pattern and the total repeat length, reflecting the CGG/AGG substructure. The result score, named *allelic score*, was calculated separately for each allele as follows:

$$A\,l\,l\,e\,l\,i\,c\,s\,c\,o\,r\,e=\left(\sum_{i=1}^{n}R_{i}\times 4^{i-1}\right)+\left(R_{n+1}\times 4^{n}\right)$$

where,

*R*_*i*_: number of CGG repeats before the first AGG interspersion of order *i*;

*i*: CGG repeat order number;

*n*: total number of AGG interspersions;

*R*_*n+*1_: number of CGG repeats after the last AGG interspersion.

Base-4 numeral system was used to ensure that the *allelic score* is unique to each of the AGG interspersion patterns and sufficiently spaced.

For the purpose of addressing allelic complexity, two different aspects of the allelic structure are considered: number of AGG interspersions and number of CGG repeats between interspersions. Higher relevance is given to the number of interspersions as, for alleles with identical number of CGG repeats, higher number of AGG interspersions is usually linked with allelic stability (Maia et al., 2016; Manor et al., 2019). As example, an allele with two AGGs shows an *allelic score* of 193 whereas an allele with a similar length but only one AGG has an *allelic score* of 59.

$$A\,l\,l\,e\,l\,i\,c\,s\,c\,o\,r\,e\,\left[{\left(\text{CGG}\right)}_{9}\,\text{AGG}\,{\left(\text{CGG}\right)}_{10}\,\text{AGG}\,{\left(\text{CGG}\right)}_{9}\right]=$$

$$\left[\left(9\times 4^{1-1}\right)+\left(10\times 4^{2-1}\right)\right]+\left(9\times 4^{2}\right)=193$$

$$A\,l\,l\,e\,l\,i\,c\,s\,c\,o\,r\,e\,\left[{\left(\text{CGG}\right)}_{10}\,\text{AGG}\,{\left(\text{CGG}\right)}_{19}\right]=$$

$$\left(19\times 4^{1-1}\right)+\left(10\times 4^{1}\right)=59$$

This mathematical model is protected with a national patent (reference – 115244) and international patent application submitted on december 6, 2019 (reference – PCT/IB2019/060520).

### Application and Validation of the Mathematical Model

*Allelic scores* ranged from 15 to 825 (cohort 1) and 15 to 828 (cohort 2), with most samples scoring below 220 (95.4%) and six with a score in the order of 800, due to the presence of three AGG interspersions (Tables 1, 2). Scores under 220 either represent zero, one or two AGG interspersions; above two AGG interspersions, the *allelic score* grows exponentially. An exploratory cluster analysis identified four major clusters, with observations within each quadrant separated in both axes by an *allelic score* of 150 (**Supplementary Figures 1, 2**). Similar behaviors were observed among the two quadrants where *allelic scores* were both lower than 150 or both higher than 150, and the other two where alleles show low and high *allelic score*, allowing the definition of two groups. The *equivalent* group contains samples where both alleles show a similar complexity, and the *dissimilar* group with samples where alleles show a different complexity. These groups include samples with three AGGs as the behavior of their alleles fits that of other samples in the same quadrant (**Supplementary Figures 1, 2**). In both groups, an exponential model was used to describe the correlation between the *allelic score* of each allele. Significant correlations were found: cohort 1 – *equivalent* group: *r* = 0.8092; df = 24; *p* < 0.0001 and *dissimilar* group: *r* = −0.7067; df = 22; *p* < 0.0001 (**Supplementary Figure 3**). To validate the mathematical models and their reproducibility, a covariance (ANCOVA) analysis was used to compare the models calculated for cohort 1 and the same models computed using cohort 2 data (*equivalent* group: *r* = 0.8603; df = 43; *p* < 0.0001 and *dissimilar* group: *r* = −0.8716; df = 33; *p* < 0.0001) (**Supplementary Figure 4**). There was no statistically significant difference between cohort 1 (development cohort) and cohort 2 (validation cohort) with respect to the *equivalent* and *dissimilar* group’s models, as demonstrated by the coincident regression lines (**Supplementary Figure 5**). A more robust model including all observations (both cohorts) was derived: *equivalent* group – *F*_(2, 68)_ = 1.8048; *p* = 0.1723: ln(score 2) = 3.6452 + 0.0088 × score 1 and *dissimilar* group – *F*_(2, 55)_ = 0.9574; *p* = 0.3902: ln(score 2) = 5.6944 – 0.0065 × score 1.

Seven samples from each group (cohort 2) were tested for XCI pattern (**Supplementary Table 1**). Interestingly, in a sample belonging to the *dissimilar* group, *FMR1* mPCR showed extreme skewing (85%) toward the smallest “low zone” allele.

## Discussion

Our study focused on developing a tool to score and evaluate the complexity of the *FMR1* gene repetitive tract structure. To this end, a mathematical model was designed that computes the *FMR1* gene CGG repeat length, as well as the AGG interspersion number and pattern. The output, a number designated *allelic score*, deciphers a functional model to predict the complexity of allele combinations. Two cohorts of young, healthy, and potentially fertile females were used independently for development and validation studies. The fact that two statistically significant groups, *equivalent* and *dissimilar*, were identified in both cohorts, justified the pooling of data. Furthermore, the identification of two groups shows the model’s ability to compare the complexity of the two alleles. Interestingly, the *dissimilar* group is enriched with “low zone” heterozygous samples (herein defined as CGG ≤ 26). It has been proposed that these “low zone” alleles may exert negative effects, although controversial (Spitzer et al., 2012; Mailick et al., 2014; Gleicher et al., 2015; Ruth et al., 2016). Another study claims that normal *FMR1* repeat length outside 26 > CGG > 34 concur with a higher XCI skew, a putative mechanism underlying the ovarian reserve impairment (as assessed by AMH), particularly in infertile older females (Barad et al., 2017). Moreover, the AGG “protective” effect toward a decreased risk of ovarian malfunction was observed in females carrying premutated alleles with two or more interspersions (Lekovich et al., 2018). According to our model, these alleles would show a high *allelic score*, which seems to suggest a correlation between the allelic complexity and a protective effect. Replication of these results is still required using larger control and patient cohorts. Nonetheless, with this mathematical model developed to calculate the *FMR1 allelic score*, further research can now be undertaken with a different perspective in terms of *FMR1* characterization.

## Data Availability Statement

The original contributions presented in the study are included in the article/supplementary material, further inquiries can be directed to the corresponding author.

## Ethics Statement

The studies involving human participants were reviewed and approved by the Ethics Committe of Centro Hospitalar Universitário do Porto. Written informed consent from the participants was obtained in accordance with the national legislation (lei 12/2005) and the institutional requirements. The participants provided their written informed consent to participate in this study.

## Author Contributions

PJ conceived and designed the study together with BR. AN developed the mathematical model and performed the statistical analysis with BR who also carried out laboratory work, analyzed the data, and drafted the manuscript. FS performed methylation/inactivation studies. EV-F, NM, IM, and RS provided critical feedback, helped conduct the research, and contributed toward the manuscript. All authors discussed the final results and critically reviewed the manuscript.

## Conflict of Interest

The authors declare that the research was conducted in the absence of any commercial or financial relationships that could be construed as a potential conflict of interest.
